# Supplementary material for: A topological data analytic approach for discovering biophysical signatures in protein dynamics
Source: PLoS Comput Biol. 2022 May 2;18(5):e1010045. doi: 10.1371/journal.pcbi.1010045 (PMC9098046; doi:10.1371/journal.pcbi.1010045)
Supplement: S1 Table — Each entry represents the time (in seconds) it takes to run each step of the SINATRA Pro algorithm based on: (i) the total number of proteins analyzed N = 50, (ii) the number of cones of directions c = {15, 20}, (iii) the number of directions within each cone d = {4, 8}, and (iv) the number of sublevel sets (i.e., filtration steps) used to compute the Euler characteristic (EC) along a given direction l = {25, 50}. We simulate 10 different datasets for each combination of parameter values. Values appearing after the ± symbol are the standard deviations of these estimated times across the different runs. Each analysis was performed using simulated protein structures with ∼2700 atoms and all runtimes were computed using a central processing unit (CPU) with 8 cores and 128 gigabytes (GB) of RAM. (PDF) [file pcbi.1010045.s029.pdf]

Total Proteins $N = 50$	Number of Cones $c = 15$			
	Directions per Cone $d = 4$		Directions per Cone $d = 8$	
	Sublevel Sets $l = 25$	Sublevel Sets $l = 50$	Sublevel Sets $l = 25$	Sublevel Sets $l = 50$
(1) Read in PDB Structures	45.0 ± 0.6	45.0 ± 0.5	44.6 ± 0.0	45.0 ± 0.5
(2) Construct Meshes/Simplicial Complexes	79.0 ± 1.2	78.1 ± 0.2	78.1 ± 0.6	78.3 ± 0.4
(3) Compute Diff. Euler Characteristics	74.1 ± 0.4	74.5 ± 0.2	86.0 ± 0.7	86.6 ± 0.4
(4) Compute Atomic Variable Importance	85.0 ± 11.5	128.3 ± 3.1	126.1 ± 4.5	461.5 ± 10.8
(5) Reconstruct PDB Structures/Enrichments	22.7 ± 0.4	22.7 ± 0.1	23.0 ± 0.2	23.4 ± 0.4
**Total Runtime:**	$\mathbf{305.9 \pm 11.6}$	$\mathbf{348.6 \pm 3.1}$	$\mathbf{357.8 \pm 4.6}$	$\mathbf{694.7 \pm 0.9}$

Total Proteins $N = 50$	Number of Cones $c = 20$			
	Directions per Cone $d = 4$		Directions per Cone $d = 8$	
	Sublevel Sets $l = 25$	Sublevel Sets $l = 50$	Sublevel Sets $l = 25$	Sublevel Sets $l = 50$
(1) Read in PDB Structures	45.1 ± 0.6	45.5 ± 0.7	45.2 ± 0.7	44.9 ± 0.5
(2) Construct Meshes/Simplicial Complexes	85.1 ± 13.1	78.7 ± 0.8	81.4 ± 5.8	77.8 ± 0.5
(3) Compute Diff. Euler Characteristics	79.4 ± 0.3	80.3 ± 1.3	93.2 ± 0.5	93.7 ± 0.4
(4) Compute Atomic Variable Importance	82.3 ± 0.3	192.3 ± 3.2	195.6 ± 3.4	991.2 ± 7.4
(5) Reconstruct PDB Structures/Enrichments	22.8 ± 0.2	23.1 ± 0.5	23.4 ± 0.4	23.3 ± 0.3
**Total Runtime:**	$\mathbf{314.8 \pm 13.2}$	$\mathbf{420.0 \pm 3.7}$	$\mathbf{438.7 \pm 6.8}$	$\mathbf{1230.9 \pm 7.5}$
