# Supplementary material for: A topological data analytic approach for discovering biophysical signatures in protein dynamics
Source: PLoS Comput Biol. 2022 May 2;18(5):e1010045. doi: 10.1371/journal.pcbi.1010045 (PMC9098046; doi:10.1371/journal.pcbi.1010045)
Supplement: S2 Table — Each entry represents the time (in seconds) it takes to run each step of the SINATRA Pro algorithm based on: (i) the total number of proteins analyzed N = 100, (ii) the number of cones of directions c = {15, 20}, (iii) the number of directions within each cone d = {4, 8}, and (iv) the number of sublevel sets (i.e., filtration steps) used to compute the Euler characteristic (EC) along a given direction l = {25, 50}. We simulate 10 different datasets for each combination of parameter values. Values appearing after the ± symbol are the standard deviations of these estimated times across the different runs. Each analysis was performed using simulated protein structures with ∼2700 atoms and all runtimes were computed using a central processing unit (CPU) with 8 cores and 128 gigabytes (GB) of RAM. (PDF) [file pcbi.1010045.s030.pdf]

Total Proteins $N = 100$	Number of Cones $c = 15$			
	Directions per Cone $d = 4$		Directions per Cone $d = 8$	
	Sublevel Sets $l = 25$	Sublevel Sets $l = 50$	Sublevel Sets $l = 25$	Sublevel Sets $l = 50$
(1) Read in PDB Structures	52.2 ± 0.3	50.6 ± 0.5	50.4 ± 0.1	50.4 ± 0.1
(2) Construct Meshes/Simplicial Complexes	159.7 ± 2.1	158.7 ± 0.6	157.8 ± 1.1	157.6 ± 0.8
(3) Compute Diff. Euler Characteristics	151.8 ± 0.3	152.2 ± 0.7	178.1 ± 0.9	178.4 ± 0.6
(4) Compute Atomic Variable Importance	89.5 ± 0.7	141.2 ± 1.5	141.8 ± 4.8	489.5 ± 3.4
(5) Reconstruct PDB Structures/Enrichments	46.3 ± 0.3	46.5 ± 0.2	46.8 ± 0.1	47.2 ± 0.3
**Total Runtime:**	**499.5 ± 2.3**	**549.2 ± 1.8**	**574.9 ± 5.0**	**923.1 ± 3.6**

Total Proteins $N = 100$	Number of Cones $c = 20$			
	Directions per Cone $d = 4$		Directions per Cone $d = 8$	
	Sublevel Sets $l = 25$	Sublevel Sets $l = 50$	Sublevel Sets $l = 25$	Sublevel Sets $l = 50$
(1) Read in PDB Structures	50.4 ± 0.1	50.3 ± 0.1	50.3 ± 0.1	50.4 ± 0.2
(2) Construct Meshes/Simplicial Complexes	158.1 ± 0.4	157.8 ± 1.1	157.9 ± 0.5	157.0 ± 0.9
(3) Compute Diff. Euler Characteristics	162.7 ± 0.6	162.5 ± 0.4	193.4 ± 0.8	193.6 ± 0.6
(4) Compute Atomic Variable Importance	98.1 ± 0.5	211.3 ± 2.1	209.4 ± 1.7	1022.8 ± 17.4
(5) Reconstruct PDB Structures/Enrichments	46.8 ± 0.2	46.7 ± 0.3	47.4 ± 0.2	47.6 ± 0.8
**Total Runtime:**	**516.1 ± 0.9**	**628.6 ± 2.4**	**658.4 ± 1.9**	**1471.4 ± 17.5**
