# Supplementary material for: A topological data analytic approach for discovering biophysical signatures in protein dynamics
Source: PLoS Comput Biol. 2022 May 2;18(5):e1010045. doi: 10.1371/journal.pcbi.1010045 (PMC9098046; doi:10.1371/journal.pcbi.1010045)
Supplement: S3 Table — Each entry represents the time (in seconds) it takes to run each step of the SINATRA Pro algorithm based on: (i) the total number of proteins analyzed N = 200, (ii) the number of cones of directions c = {15, 20}, (iii) the number of directions within each cone d = {4, 8}, and (iv) the number of sublevel sets (i.e., filtration steps) used to compute the Euler characteristic (EC) along a given direction l = {25, 50}. We simulate 10 different datasets for each combination of parameter values. Values appearing after the ± symbol are the standard deviations of these estimated times across the different runs. Each analysis was performed using simulated protein structures with ∼2700 atoms and all runtimes were computed using a central processing unit (CPU) with 8 cores and 128 gigabytes (GB) of RAM. (PDF) [file pcbi.1010045.s031.pdf]

Total Proteins $N = 200$	Number of Cones $c = 15$			
	Directions per Cone $d = 4$		Directions per Cone $d = 8$	
	Sublevel Sets $l = 25$	Sublevel Sets $l = 50$	Sublevel Sets $l = 25$	Sublevel Sets $l = 50$
(1) Read in PDB Structures	63.5 ± 1.3	63.3 ± 1.6	64.3 ± 2.8	63.1 ± 1.1
(2) Construct Meshes/Simplicial Complexes	305.3 ± 1.4	304.6 ± 2.2	305.4 ± 0.9	304.2 ± 1.1
(3) Compute Diff. Euler Characteristics	296.1 ± 0.8	297.1 ± 0.4	347.8 ± 1.7	348.0 ± 1.2
(4) Compute Atomic Variable Importance	111.9 ± 0.8	158.8 ± 0.7	161.8 ± 4.9	493.3 ± 1.1
(5) Reconstruct PDB Structures/Enrichments	89.1 ± 0.5	89.8 ± 1.2	90.7 ± 0.5	90.9 ± 0.3
**Total Runtime:**	$\mathbf{865.9 \pm 2.2}$	$\mathbf{913.5 \pm 3.1}$	$\mathbf{970.1 \pm 6.0}$	$\mathbf{1299.5 \pm 2.3}$

Total Proteins $N = 200$	Number of Cones $c = 20$			
	Directions per Cone $d = 4$		Directions per Cone $d = 8$	
	Sublevel Sets $l = 25$	Sublevel Sets $l = 50$	Sublevel Sets $l = 25$	Sublevel Sets $l = 50$
(1) Read in PDB Structures	63.7 ± 1.2	63.5 ± 1.5	63.9 ± 1.1	64.0 ± 1.1
(2) Construct Meshes/Simplicial Complexes	310.5 ± 11.8	304.5 ± 0.6	304.8 ± 0.9	304.7 ± 1.1
(3) Compute Diff. Euler Characteristics	320.0 ± 0.5	319.6 ± 0.4	377.0 ± 1.4	377.6 ± 1.5
(4) Compute Atomic Variable Importance	119.9 ± 0.5	226.4 ± 0.5	227.5 ± 2.3	1001.7 ± 6.9
(5) Reconstruct PDB Structures/Enrichments	91.2 ± 0.7	90.6 ± 0.7	92.8 ± 1.1	92.6 ± 1.2
**Total Runtime:**	$\mathbf{905.3 \pm 11.9}$	$\mathbf{1004.6 \pm 1.9}$	$\mathbf{1066.1 \pm 3.3}$	$\mathbf{1840.6 \pm 7.3}$
